# Developing evidence-based resources for evaluating postgraduate trainees in the biomedical sciences

**DOI:** 10.1371/journal.pone.0278297

**Published:** 2022-12-13

**Authors:** Jacqueline E. McLaughlin, Rebekah L. Layton, Paul B. Watkins, Robert A. Nicholas, Kim L. R. Brouwer

**Affiliations:** 1 Division of Practice Advancement and Clinical Education, Center for Innovative Pharmacy Education and Research, UNC Eshelman School of Pharmacy, University of North Carolina at Chapel Hill, Chapel Hill, North Carolina, United States of America; 2 Office of Graduate Education, School of Medicine, University of North Carolina at Chapel Hill, Chapel Hill, North Carolina, United States of America; 3 Division of Pharmacotherapy and Experimental Therapeutics, UNC Eshelman School of Pharmacy, University of North Carolina at Chapel Hill, Chapel Hill, North Carolina, United States of America; 4 Department of Pharmacology, School of Medicine, University of North Carolina at Chapel Hill, Chapel Hill, North Carolina, United States of America; Graduate Career Consulting LLC, UNITED STATES

## Abstract

Postgraduate trainees elevate the academic strength of institutions by conducting research, promoting innovation, securing grant funding, training undergraduate students, and building alliances. Rigorous and systematic program evaluation can help ensure that postgraduate training programs are achieving the program’s intended outcomes. The purpose of this project was to develop evidence-based evaluation tools that could be shared across federally funded biomedical training programs to enhance program evaluation capacity. This manuscript describes the evidence-based process used to determine program evaluation needs of these programs at a research-intensive university. Using a multi-phased sequential exploratory mixed methods approach, data were collected from trainees, employers, leaders, and program directors. Data analyses included document analysis of program plans, inductive coding of focus groups and interviews, and descriptive analysis of surveys. Two overarching categories–Trainee Skills and Program Characteristics—were identified including six themes each. Program directors prioritized communication, social and behavioral skills, and collaboration as the trainee skills that they needed the most help evaluating. Furthermore, program directors prioritized the following program characteristics as those that they needed the most help evaluating: training environment, trainee outcomes, and opportunities offered. Surveys, interview scripts, and related resources for the categories and themes were developed and curated on a publicly available website for program directors to use in their program evaluations.

## Introduction

Postgraduate trainees play a critical role in the success of academic institutions [1, 2]. Under the guidance of mentors who help develop their intellectual independence, postgraduate trainees elevate the academic strength of institutions by conducting research, promoting innovation, training and mentoring undergraduate students, securing grant funding, and building alliances [1–3]. In the United States, biomedical sciences doctoral students typically complete their coursework and research in 4–6 years, whereas postdoctoral trainees (colloquially known as “postdocs”) typically complete 1–5 years of activities that promote scholarly autonomy, disciplinary specialization, and entrepreneurial skills [3–6].

Recent research highlights the complexity of postgraduate career and professional development and raises concerns about the extent to which training programs align with the needs of trainees, employers, and society [1, 2, 7–9]. Career outcomes can vary widely, with biomedical trainees transitioning into various academic and non-academic careers [10, 11]. Furthermore, recent research from the NIH Broadening Experience in Scientific Training (BEST) consortium suggests that participating in professional development, such as career panels, skill-building workshops, job search workshops, site visits, and internships, neither reduces efficiency (i.e., no delayed time to degree) nor productivity (i.e., no fewer total or first author publications) [12]. Despite initial attempts to gain stakeholder perspectives [13], more research is needed to better develop and align postgraduate training with trainee goals, employer needs, entrepreneurship, and economic workforce predictions.

Nationally, this reality has been recognized across funding agencies in numerous ways. Federally funded postgraduate training programs (e.g., T32 and R25 programs) are intended to further develop and enhance scientific training and help trainees become more competitive for the job market. While goals typically vary from program to program, well-designed curricula should increase trainee competitiveness for peer-reviewed research funding, strengthen trainee publication records, and foster institutional environments conducive to success in the biomedical and health sciences [14]. Ensuring that these programs achieve their intended outcomes is crucial for promoting the success of postgraduate trainees and their universities.

As a requirement for funding, National Institute of General Medicine Science (NIGMS)-funded training programs must conduct ongoing evaluations to monitor the success of the training and mentoring activities, with an expectation that findings are disseminated to the broader community [15]. In 2020, NIGMS published a Notice of Special Interest regarding availability of funds for Administrative Supplements to Enhance Program Evaluation Capacity at institutions with NIGMS training programs [16]. If programs are not identifying training goals when creating their programing plans, it is unlikely they will be able to meet the needs of the defined outcomes, and without measuring defined outcomes, it becomes impossible to quantify the effectiveness of the proposed programming. Therefore, the emphasis of evidence-based program evaluation can support the creation of more effective programs–as well as the dissemination of program aspects that are successful and could be replicated elsewhere.

The University of North Carolina (UNC) has a rich history of NIGMS training programs across multiple schools and departments, currently including two T32 programs for postdoctoral trainees, ten T32 programs for predoctoral trainees, and one R25 for predoctoral trainees. These programs are bolstered by a strong culture of collaboration and coordination, such as the Biological and Biomedical Sciences Program (BBSP), which serves as a single admission portal and first-year program for 15 UNC graduate programs and enables students to join predoctoral T32 programs once they become eligible [17]. The breadth of T32/R25 training programs and related institutional infrastructure makes UNC ideal for systematically evaluating these programs. However, uniform processes were not in place for evaluating trainees, mentors, and program effectiveness at the institution, and suitable evaluation tools were not readily available when UNC received the NIGMS Administrative Supplement. The purpose of this project was to develop evidence-based evaluation tools that could be shared across these T32/R25 training programs to enhance program evaluation capacity. This manuscript describes the evaluation process and embedded mixed methods study utilized to understand the evaluation needs of UNC T32/R25 programs and develop related evaluation support for program directors.

## Materials and methods

**Fig 1:**
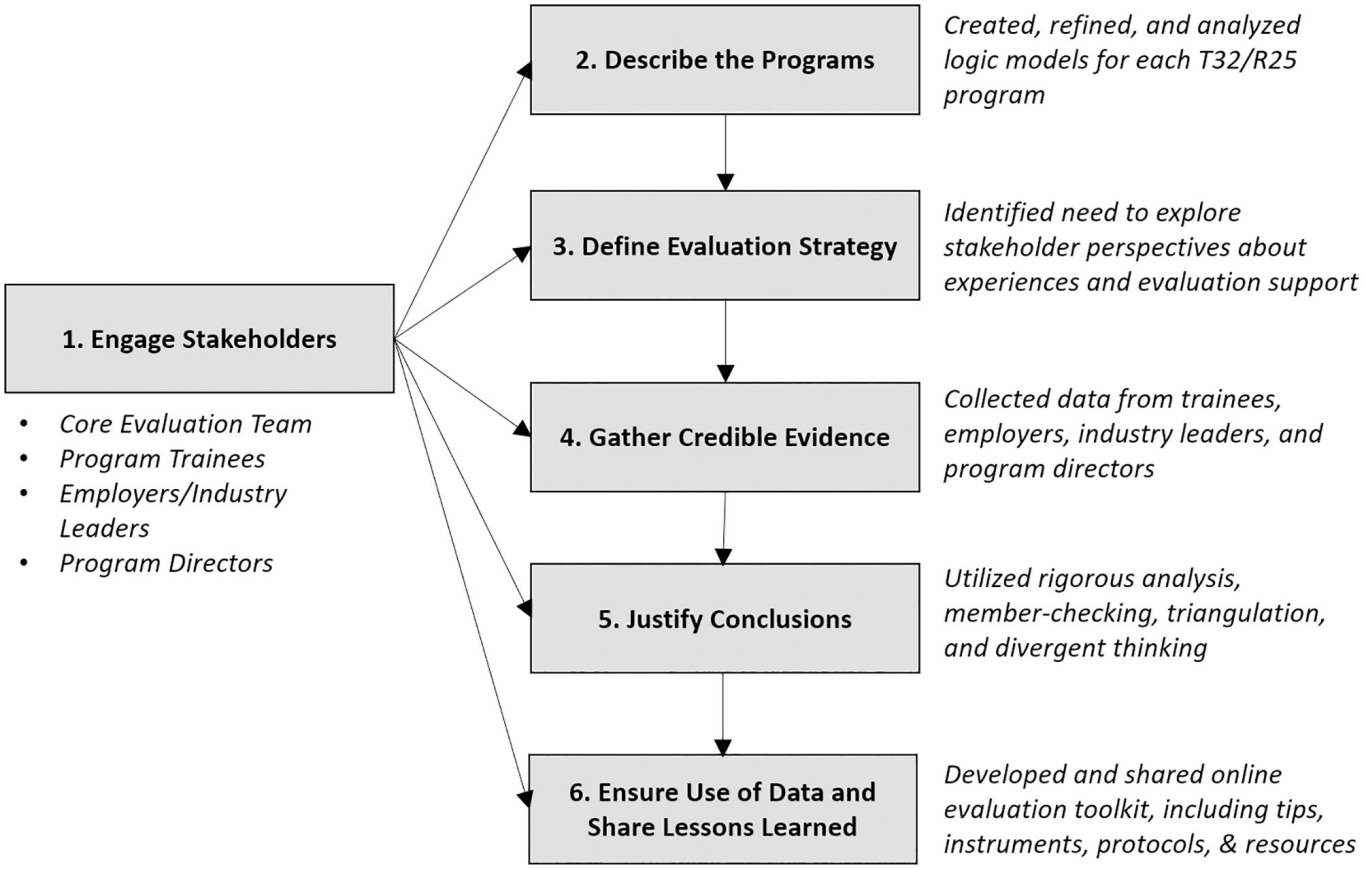
Evaluation process for determining evaluation needs for T32/R25 training programs.

**Fig 2:**
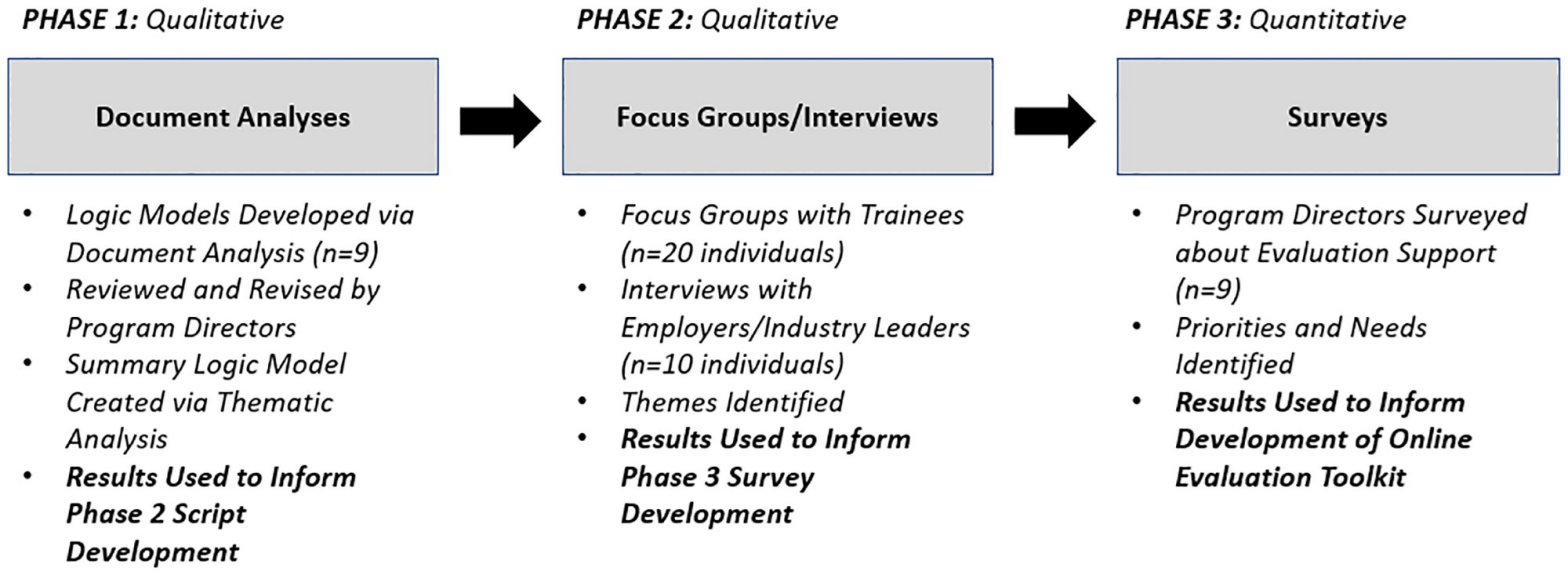
Multiphase sequential mixed methods design for determining evaluation needs of T32/R25 training programs.

As seen in Fig 1, the project team followed the evaluation framework described by the Centers for Disease Control and Prevention (CDC) Evaluation Working Group [18]. While the framework steps are interdependent and typically implemented linearly, some steps (e.g., engage stakeholders) were visited repeatedly. Portions of our process that involved systematic data collection and analysis followed a multi-phased sequential exploratory mixed methods design (Fig 2).

At the start of this project, **(1) an evaluation team was convened**, comprised of several key stakeholders: three T32 program directors [KLRB, RAN, PBW], two education specialists (1 from the UNC Eshelman School of Pharmacy [JEM] and one from the UNC School of Medicine Office of Graduate Education [RLL]), and one T32 program administrator. Team members were chosen based on the expertise needed to achieve the goals of the project. The evaluation team met approximately every two weeks to discuss progress and critical decisions. In addition, an evaluation consultant was engaged periodically throughout the process. These individuals were instrumental for providing feedback about goals, needs, process, and impact.

To better understand and **(2) describe the programs** included in this project, the evaluation team used individual T32 program plans to develop a logic model for each program. Logic models represent shared relationships among program resources, activities, outputs, and outcomes [19, 20]. Graphically, these models detail the process by which we believe the program meets its long-term outcomes (i.e., program theory): *if we have [inputs]*, *we can do [activities]*, *which will result in [outputs] that impact [outcomes]*. Nine logic models were developed by a single researcher (JEM) utilizing the T32 program plan provided by each program director. The draft logic model was provided to the program director for review and revised based on feedback. Once all nine logic models were complete, an aggregate logic model was created, reviewed, and revised by the evaluation team to reflect common elements across all programs.

With a better understanding of the training programs, the evaluation team was able to identify issues that were relevant to training program plans, articulate questions and items that remained unclear, and **(3) define an evaluation strategy** focused on relevant users, uses, and needs. Of note, the evaluation team wanted more insight into the evaluation needs of training programs from various stakeholder perspectives. As such, an evaluation strategy was developed that included data collection from trainees, program directors, employers, and industry leaders.

During the next phase, **(4) credible evaluation data were gathered** via trainee focus groups to better understand their experiences in these programs–an important issue that was not sufficiently uncovered in the document analysis. Purposive sampling was utilized to identify current training program students and postdocs, and participants were recruited via email. Four 1-hour focus groups were conducted in Fall 2020 with 20 graduate students and postdoctoral trainees from multiple training programs. Example script questions included: *What aspect of your program do you feel all doctoral/postdoctoral trainees in your program gain value from participating in*? and *What challenges have you experienced as part of your training*?

Next, interviews were conducted with select employers of training program alumni and industry leaders, as these stakeholders are well-positioned to provide insight into needs and gaps in the field. Purposive sampling was utilized, and participants included Dean-level academic leaders, former federal program officers, leadership of professional organizations, and employers from Clinical Research Organizations (CRO), pharmaceutical industry, biotechnology companies, and regulatory agencies. Participants were recruited via email and 1-hour interviews, which were conducted in December 2021 and January 2022 (n = 10). Example script questions included: *What are appropriate program metrics that should be included in a program evaluation*, *from your perspective*? and *What changes do you see in your industry in the next 5–10 years that we should be considering*? All focus group and interview sessions were conducted via Zoom with at least one interviewer and one note-taker.

Sessions were recorded using field notes that were inductively coded independently for themes by two researchers. This qualitative analysis approach enabled the researchers to identify semantic themes, provide a description of what existed in the data, and allowed for the data to be organized into interpretative patterns (i.e., categories, themes). Saturation of themes was achieved, providing a rich description of the entire data set, and themes were pervasive across participants. Achieving data saturation indicated that the sample size was sufficient for the intended purpose (i.e., additional interviews were not necessary) and that findings were relevant regardless of participant role (e.g., Dean, CRO). Categories and related themes were refined, and discrepancies were resolved via consensus-building discussions [21, 22].

Using the resulting categories and themes from the focus groups and interviews, program directors were surveyed about their evaluation priorities and needs. The survey was administered to the 11 NIGMS Program Directors with one reminder email during Spring 2022. The survey introduction described the purpose of the study and how the themes and categories were developed. For each category, participants were asked to *rate* *how important you think each of the [themes] are for evaluating your T32/R25 training program* (1-Not At All Important to 5-Extremely Important), and to *rank order* *the [themes] that you would most like help with evaluating in your T32/R25 training program* (1-Least Help to 6-Most Help). An open text field invited additional items that participants wanted help evaluating. Data were analyzed using descriptive statistics (mean ± standard deviation (SD)).

Taken together, the logic models, focus groups, interviews, and surveys provided rich data and relevant insight into the evaluation needs of the training programs. This multiphase process enabled us to **(5) justify conclusions** through member-checking, triangulation, divergent thinking, and disclosure of limitations. For example, in April 2021 the members of the evaluation team met with the program directors to review and discuss findings from the survey with the hopes of further understanding their evaluation needs (i.e., member-checking). This discussion and feedback, in turn, enabled the evaluation team to identify and develop a new online evaluation toolkit comprised of educational resources and tips about evaluation, protocols and instruments for data collection, key findings from this work, related summary information about core training skills, and contact information for evaluation experts. In December 2021, the core evaluation team met with program directors via zoom to share and discuss the online evaluation toolkit. This positioned us to **(6) ensure use of evaluation data and share lessons learned**.

### Ethical considerations, consent

This project was submitted to the UNC Institutional Review Board (#18–3140) and was determined to be non-human subjects research according to 45 CFR 46.104. Due to this classification, formal consent was not required; however, study communications included information about participation via email invitations and verbal reminders, such as prior to beginning each focus group or interview, with reminders that participation was voluntary, and that all data would be de-identified prior to sharing.

## Results

**Fig 3:**
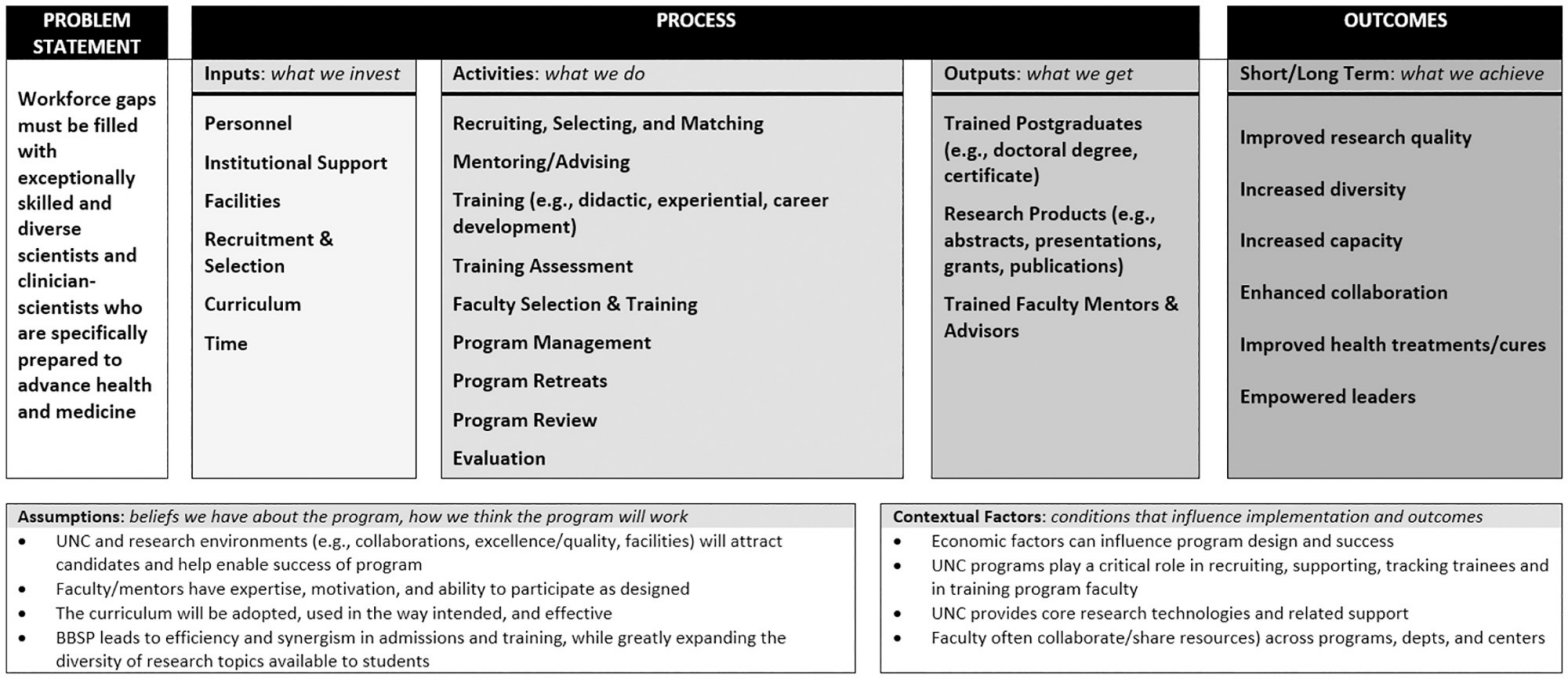
Abbreviated logic model depicting summary inputs, activities, outputs, and outcomes of UNC T32/R25 training programs.

Fig 3 depicts an abbreviated version of the aggregate logic model (*NOTE*: *the full logic model can be found online at* https://tarheels.live/t32programevaluation/logic-models/). Of note, the logic model highlights common elements across all included T32/R25 programs, as well as an overarching goal of addressing workforce gaps with *exceptionally skilled and diverse scientists and clinician-scientists who are specifically prepared to advance health and medicine* (Fig 3). Common inputs included personnel, institutional support and infrastructure, and time. Common activities addressed needs for trainees (e.g., recruitment, selection, training, assessment), faculty (e.g., selection, training), and the program (e.g., management, retreats, evaluation). Common outputs consisted of trained personnel (e.g., trainees, faculty mentors) and research products (e.g., publications, grants) leading to various outcomes, such as improved research quality, increased diversity, increased capacity, enhanced collaboration, and ultimately, advances in healthcare and the next generation of leaders. From the logic models, focus group and interview questions were drafted to learn more about the training and assessment activities.

In the focus groups and interviews, two overarching categories were identified related to needs in postdoctoral training: trainee skills and program characteristics (Table 1). Trainee skills included themes of communication (e.g., written, oral, scientific, interpersonal), social and behavioral skills (e.g., adaptability, resilience), collaboration, career awareness/development (e.g., business acumen, job market readiness), discipline-specific skills (e.g., technical skills), and productivity. Program characteristics included themes of training environment (e.g., work-life balance, quality of mentoring), trainee outcomes (e.g., job placement), opportunities offered (e.g., funding for professional development, mentor selection/matching), trainee productivity (e.g., number and types of presentations, publications, grants), program transparency (e.g., clarity regarding selection of trainees and mentors, mentor selection/matching), and assessment culture (e.g., interviews/focus groups, surveys).

**Table 1:** Results from focus groups, interviews, and *program* director surveys, including categories, themes, and the support needed for evaluation.

Category	Theme	Examples	Example Quote	Importance to PD* (n = 9) Mean ± SD	Support Needed by PD** (n = 9) Mean ± SD
***Trainee Skills***	Communication Skills	Written, oral, conflict resolution, client-facing, receiving feedback	*Effective communication skills really make a difference in the long-term career trajectory*. *These skills can make or break a career*. (Employer/Leader)	4.43 ± 0.49	4.23 ± 0.75
	Social & Behavioral Skills	Adaptability, initiative, negotiation, resilience, well-being	*Employers are asking for intangible personal soft skills needed to be successful in companies*, *like resilience*, *communication*, *persistence*. (Leader)	3.71 ± 0.88	2.83 ± 1.77
	Collaboration Skills	Interpersonal, teamwork, community building	*Trainees need to be experts in their own disciplines*, *but [also be] boundary crossers and able to work across disciplines*. (Employer/*Leader*)	4.29 ± 0.45	2.67 ± 1.25
	Career Awareness Skills	Business acumen, career exploration, career *preparation*, market readiness	*We need exposure outside of academia earlier in the program*. (Trainee)	4.14 ± 0.64	2.50 ± 1.12
	Discipline-Specific Skills	Analytical thinking, knowledge expertise, problem solving, technical skills	*As more aspire to careers outside of academia*, *competencies become more important…are trainees gaining competencies that they need for THEIR career choice*? (Employer/*Leader*)	4.43 ± 0.73	1.77 ± 1.11
	Productivity Skills	Completing projects, meeting deadlines, preparing presentations	*Practice is the most important thing when it comes to science*. (Trainee)	4.43 ± 0.73	1.00 ± 1.83
***Program Characteristics***	Training Environment	Work-life balance, quality of mentoring, peer community institutional support, satisfaction	*Mentors don’t always understand that they can change the course of a student’s path*. (Trainee)	*4*.*22 ± 0*.*63*	4.11 ± 1.29
	Trainee Outcomes	Job placement, number of job offers, met professional goals, fellowships, alumni involvement	*Are they satisfied with the careers once they go into them*? *Related to that is underemployment…are they utilizing their skills and whether they feel they want more related to career satisfaction*? (Employer/Leader)	4.44 ± 0.68	2.89 ± 1.03
	Opportunities Offered	Funding for training/travel, mentor selection and matching, learning contexts (e.g., lab, networking)	*Being more well-funded means less stress we have about things that aren’t [related to our research]*. (Trainee)	4.22 ± 0.63	2.56 ± 1.17
	Trainee Productivity	Number/types of presentations, publications, awards	*Each program should have core metrics related to the goals of the training grants*, *for example pubs & presentations & conferences*, *workshops & local presentations*. (Trainee)	4.22 ± 0.79	1.89 ± 1.85
	Program Transparency	Clarity about how trainees and mentors are selected and matched, program successes	*I didn’t understand the importance of funding and how it works…how much time and money would be provided for travel*? *Which PIs get money and which students receive funding*? (Trainee)	3.75 ± 0.66	1.78 ± 1.03
	Assessment Culture	Focus groups, surveys, stakeholder engagement (e.g., T32 Director, Alumni)	*Focusing more on getting the perceptions of students/trainees*, *alumni*, *and employers…*.*find key gaps or what you wish you would’ve learned in our program*. (Employer/Leader)	3.67 ± 0.66	1.78 ± 1.62

PD = program director; SD = standard deviation

* Program directors were asked to *rate how important you think each of the [training skills* or *program characteristics] are for evaluating your T32/R25 training program*, from (1-Not At All Important to 5-Extremely Important)

**Program directors were asked to *rank order the [training skills* or *program characteristics] that you would most like help with evaluating in your T32/R25 training program*, from (1-Least Help to 6-Most Help)

In addition, focus group and interview participants provided various suggestions and ideas regarding assessment strategies that could help elucidate key aspects of their program. This compiled list included: measurement instruments (e.g., surveys, focus groups, interviews/exit interviews); evaluation focus (e.g., applicant characteristics, trainee evaluations, mentor evaluations, post-program career progression, program efficacy); and program metrics (e.g., number of participants, graduates, research products, job placement, trainee perceptions, and employer perceptions).

When asked about the importance of the trainee skills identified in the focus groups and interviews (1-Not At All Important to 5-Extremely Important), program directors (n = 9, 81.81% response rate) rated communication as most important (4.43 ± 0.49), followed by discipline-specific skills (4.43± 0.73), and productivity skills (4.43 ± 0.73) (Table 1). Program directors ranked (1- Least Help to 6-Most Help) communication as the item they needed the most support evaluating (4.23 ± 0.75), followed by social and behavioral skills (2.83 ± 1.77), and collaboration (2.67 ± 1.25). Communication was clearly prioritized by program directors in terms of importance and support needed.

When asked about the importance of the program characteristics identified in the focus groups and interviews, program directors rated trainee outcomes (4.44 ± 0.68), training environment (4.22 ± 0.63), opportunities offered (4.22 ± 0.63), and trainee productivity (4.22 ± 0.79) as most important (Table 1). Program directors ranked training environment (4.11 ± 1.29), trainee outcomes (2.89 ± 1.03) and opportunities offered (2.56 ± 1.17) as items they needed the most support evaluating. Training environment, trainee outcomes, and opportunities offered were clearly prioritized by program directors in terms of importance and support needed.

Survey results and subsequent discussion with the surveyed program directors were used to identify the need for data collection instruments aimed at supporting evaluation of communication, social and behavioral skills, and collaboration along with training environment, trainee outcomes, and opportunities offered. In some cases, program directors also articulated a need for scholarly resources related to skills considered challenging to address or develop (e.g., adaptability). As such, the core evaluation team developed a *core survey* for T32/R25 program evaluation that included multiple survey items related to each theme, along with 11 short (i.e., 5–10 items) *modular surveys* that each addressed a specific theme of interest to the program directors (e.g., communication, adaptability). Most modular surveys also were accompanied by a one-page evidence-based summary that provided background information, tips for trainees, tips for mentors, and resources for more information.

To promote accessibility, an online evaluation toolkit was created (https://tarheels.live/t32programevaluation/). The website included background information about evaluation, tips for logic models, downloadable surveys and related evidence-based summary resources, interview scripts and protocols, additional resources, and contact information for the core evaluation team.

## Discussion

Program evaluation is a critical process for determining the extent to which federally funded postgraduate training programs are achieving their intended outcomes, such as elevating outcomes in biomedical sciences training, making trainees more competitive for the job market, and meeting the research needs of the nation. Although NIGMS training grant proposals must include an evaluation plan, funded programs may benefit from support in designing and operationalizing their evaluations (e.g., identifying shared components across programs and supporting perceived areas of need). Using a common evaluation framework [18], we demonstrated a strategy for creating synergy and collaboration across training programs at a single institution. Specifically, the systematic process described in this manuscript helped our interdisciplinary team identify common program evaluation priorities and develop related, evidence-based evaluation tools for use across the institution. We believe this work will advance evaluation efforts and ensure that postgraduate training programs in the biomedical sciences are well-positioned to collect and utilize data to inform decision-making.

Results of this study align with a growing body of research that highlights the importance of postgraduate career skills that extend beyond traditional, discipline-specific skills. In the pharmaceutical sciences, for example, researchers have identified communication, collaboration, adaptability, flexibility and curiosity, among others, as key skills [23, 24]. Similarly, Sinche and colleagues identified written and oral communication skills, project management and creativity/innovative thinking as highly-rated transferrable skills by alumni [25]. Recently, some training programs also have focused on the development of skills related to resiliency, mental health, and wellness [26]; the ability to lead and function in diverse teams [27–29]; and communication outside disciplinary channels and audiences [30, 31]. Surprisingly, there appeared to be a gap between program director perspectives regarding the importance of Social and Behavior Skills and their need for support to evaluate these skills. As a growing body of research highlights the importance of social and behavioral skills, program directors may be simultaneously recognizing the importance of evaluating these skills but struggling to understand exactly how they impact trainee development.

Targeted career-development programming and professional-development training can assist in career placement [32] without hindering productivity or efficiency [12]. In addition to NIGMS funding requirements for career outcomes reporting, recent efforts to increase career outcomes reporting have enabled more rigorous evaluation of such outcomes [4, 10, 33–36]. A recent initiative to create more transparent access to evidence-based research on graduate and postdoctoral training was built through dozens of stakeholder insights across national organizations, indicating a true need for better program evaluation and dissemination thereof [37]. Our results indicate the need to focus on crucial program characteristics, namely training environment and opportunities offered, on trainee outcomes. Furthermore, trainee outcomes need to be more broadly defined in the research, including expanded factors beyond basic skill development, time-to-degree, publications, first job placements, and alumni job satisfaction. Although assessment culture and program transparency were identified as themes during the focus groups and interviews, they were not highly rated by program directors, which may be an important gap to explore in future research.

While the findings in this study may be transferable to other institutions, programs should give careful consideration to trainee skills and program characteristics most relevant to their program outcomes and institutional values. It is not our intent to suggest that the findings of our evaluation are the only important aspects of postgraduate training programs–only that these themes emerged from our data, collected from a select group of stakeholders at a specific point in time. Further, our analysis prioritized overarching themes at the potential expense of more specific aspects of training that may be essential for these programs. Additional aspects of postgraduate training known to affect trainee outcomes, such as equity, diversity, and inclusion (EDI), and entrepreneurship also should be considered for inclusion in program evaluations.

Several evaluation strategies utilized in this process are worth noting. Although often overlooked as an evaluation tool, logic models are critical for articulating how various elements of a program are believed to relate to one another [38]. While logic models typically include common core elements (e.g., resources, activities, outputs), they are a flexible tool that can vary by content, visual representation, and stakeholder beliefs about how a program works [39]. The logic models developed in this study enabled us to better understand individual programs, identify commonalities across programs, focus data collection on relevant activities and stakeholders, and determine next steps. However, creating a consensus logic model (Fig 3) required us to prioritize commonalities over program specifics and nuances, potentially overshadowing distinctive features of the programs included. As such, future research could explore strategies for developing more balanced consensus models and should focus specifically on relationships between various elements (e.g., activities and outputs) [40].

The engagement of the various stakeholders was also a strength of this study. The importance of stakeholders is often acknowledged, yet researchers rarely attend to stakeholder needs, concerns, priorities, and perspectives in the evaluation design [41, 42]. In our study, program directors offered input into program evaluation needs, provided feedback on key findings (i.e., member-checking), helped focus instrument development on relevant constructs, and determined next steps. Employers and industry leaders provided specific insights into contemporary needs of the workforce, while trainees provided rich descriptions of their experiences in the training programs and institution. Although existing program evaluations addressed some identified constructs and needs, we believe that the input from these stakeholders will help synergize program evaluations broadly while providing opportunities for customizing measures of interest for each program. Of the recognized stakeholder groups in evaluation [43], our study included people who have direct responsibility for the programs (e.g., program directors) and people who are the intended beneficiaries of the programs (e.g., trainees, employers). In subsequent work, program alumni also could provide useful insight.

This study has several limitations. First, this evaluation was conducted at a single, research-intensive institution with a scientifically diverse graduate student and postdoctoral population. While this design limits generalizability, the needs and experiences of these participants are likely reflected at similar types of institutions. Second, the participants interviewed were volunteers, which may have introduced self-selection bias, and did not include program alumni or every type of employer (e.g., management consultant). However, the mixed methods design of this study enabled us to collect data from various stakeholders, utilize results to inform subsequent data collection and decision-making, and demonstrate triangulation across data sources. Third, the focus was broad (across programs) at the expense of exploring specific design elements of the training programs in depth [44]. Fourth, our sample of employers was relatively small and professionally diverse. Fifth, this research focused on design and implementation, leaving the impact of the developed toolkit unknown. Subsequent evaluation efforts will include an outcomes evaluation that explores website engagement (e.g., number of times accessed, number of times the materials are downloaded) and effectiveness of the evaluation tools.

In conclusion, this work contributes to a clear need concerning program evaluation in federally funded biomedical sciences training programs and provides a model for enhancing program evaluation capacity. Institutions must invest resources in evaluation to ensure programs are designed, implemented, and enhanced effectively. Based on our experience, strategic partnerships between program directors and evaluation experts can promote critical evaluation discourse, data collection, and decision-making. We recommend that other institutions offer similar evaluation expertise and encourage collaborations that promote evaluation capacity. At our university, staff and faculty with evaluation expertise are available to provide ongoing evaluation support for these programs, individually and collectively. The results of this work informed improvements at UNC and are offered as a resource to help others support their postgraduate training programs.
